# Effects of Raw and Fermented Rapeseed Cake on Growth Performance, Methane Production, and Breast Meat Fatty Acid Composition in Broiler Chickens

**DOI:** 10.3390/ani10122250

**Published:** 2020-11-30

**Authors:** Min Gao, Adam Cieślak, Bartosz Kierończyk, Haihao Huang, Yulianri R. Yanza, Anita Zaworska-Zakrzewska, Damian Józefiak, Małgorzata Szumacher-Strabel

**Affiliations:** Department of Animal Nutrition, Poznań University of Life Sciences, Wołyńska 33, 60-637 Poznań, Poland; min.gao@up.poznan.pl (M.G.); adam.cieslak@up.poznan.pl (A.C.); bartosz.kieronczyk@up.poznan.pl (B.K.); haihao.huang@up.poznan.pl (H.H.); yulianri.yanza@up.poznan.pl (Y.R.Y.); anita.zaworska-zakrzewska@up.poznan.pl (A.Z.-Z.); damian.jozefiak@up.poznan.pl (D.J.)

**Keywords:** fermented rapeseed cake, methane emission, broiler chickens, growth performance, breast fatty acids, alternative protein source

## Abstract

**Simple Summary:**

The addition of raw rapeseed cake (RRC) to broiler feed may avoid the negative effects of soybean meal (SBM), including its genetically modified origin, environmental impact, and nonprospective flexible price. Processing RRC through fermentation has also been shown to be beneficial: adding fermented rapeseed cake (FRC) to the broiler diet is a nutritional approach that addresses both the environmental issue of broiler enteric methane emission and the economic issues of soybean meal. The aim of this study was to assess the effects of partial replacement of soybean meal by a 15% addition of RRC or FRC to the diets of broiler chickens on methane emission, nitrogen retention, ether extract digestibility, growth performance, and breast muscle fatty acid composition. The FRC did not show any unfavorable effects on growth performance or nutrient utilization, and it improved the fatty acid profile of the breast muscle; additionally, both methanogens and methane emission from the fermentation of cecal contents were significantly limited. In conclusion, FRC as a partial substituent may be a valuable alternative to soybean meal in broiler chicken nutrition.

**Abstract:**

The study was conducted to evaluate the effects of partial replacement of soybean meal (SBM) by 15% raw or fermented rapeseed cake (RRC or FRC) to broilers’ diets on growth performance, nutrient utilization, methane emission, and breast muscle fatty acid (FA) composition. A total of 420 one-day-old female Ros 308 broilers were used in three independent experiments (300 birds in the first experiment and 60 in the second and third experiments). In each trial, three treatments were set up: a control group (without rapeseed), and diets replaced soybean meal with 15% addition of RRC or FRC. Birds fed the FRC diet experienced no effect (*p* > 0.05) on performance or nutrients utilization. Methane emission and total methanogen population in the ceca was decreased (*p* < 0.05) with the FRC diet. The concentrations of n-3 and n-6 FAs in the breast tissue of fourteen-day-old birds were not affected (*p* > 0.05) by FRC. However, the n-6/n-3 ratio in the breast muscle of 28-day-old birds was reduced (*p* < 0.001). In conclusion, the replacement of SBM by FRC in the broiler diets did not show any unfavorable effects on performance or nutrient utilization. Furthermore, the breast meat FA profile was improved, methanogen counts significantly decreased, and methane emission was limited.

## 1. Introduction

Statistical estimates point to a human population of 9.7 billion being reached by 2050 [1]. This upward trend has heightened the need for food of both animal and plant origins. The amount of animal-derived protein being produced is expected to nearly double by 2050 [2]. Greater demand for animal-derived protein for human beings is associated with an increased risk of protein deficiency. Consequently, it is of great interest to find alternative sources of protein that can support animal feed production [3]. From 1961 to 2019, there was a significant increase in poultry meat production, which soared from 9 to 125 million tons. Additionally, it has been reported that poultry meat now represents about 38.2% of global meat production, and continues to rise, being projected to grow by more than 38.7% in 2028 [4].

The demand for animal protein is being addressed by rapid increases in animal stocks, which are known to play a crucial role in the production of greenhouse gases (GHGs). Animal-based foods, which involve the production of higher levels of GHGs than plant-based foodstuffs, contribute to climate change [5]. At the same time, the byproducts of oilseeds such as soy, sunflower, and rapeseed are frequently added to animals’ diets as a protein source, where they complement native protein crops. Such food additions are valued not only because of their protein content but also because of their form of administration. Recently, there has been renewed interest in rapeseed byproducts as a component of poultry feed. The use of raw rapeseed cake has many advantages, in conjunction with its relatively high concentrations of monounsaturated fatty acids (MUFAs) and low n-6/n-3 ratio. Rapeseed cake fed to broiler chickens and turkeys resulted in a lower n-6/n-3 ratio in the breast muscle. This was the result of increased n-3 fatty acids intake. The lower n-6/n-3 ratio in animal products is beneficial for consumers’ health [6,7,8].

However, raw rapeseed cake contains high levels of antinutritional factors (ANFs) such as glucosinolates and non-starch polysaccharides (NSPs), which may have an adverse effect on performance [9]. Nevertheless, refinement can increase their usability: for example, thermal processing of rapeseed can inactivate myrosinase and reduces the toxicity of glucosinolates. Literature data indicates that postextraction fermentation of rapeseed meal using bacteria and yeast (such as *Rhizopus oligosporus*, *Aspergillus oryzae*, or *Lactobacillus fermentum*) can reduce the level of aliphatic compounds (by 57.7%), indole glucosinolates (by 97.3%), oligosaccharides (by 73%), lignin and NDF (by 25%), and phytic acid (by 86%), depending on the composition of the inoculum and conditions of the process [10,11]. The available literature seems to contain no data on the use of this material in complete diets for broiler chickens, especially in the context of the size of GHG emissions; however, some promising data were published by Drażbo et al. [8,12], who have shown that the replacement of raw rapeseed cake (RRC) with fermented rapeseed cake (FRC) in diets led to an increase in the final BW of turkeys and improvements in the fatty acid profile of breast muscles.

In the scope of poultry production growth, the issue of GHG emission (mainly methane) is becoming a strategic problem, because although the amount of gas emitted by one bird is relatively low, the global total is nevertheless high. The poultry industry thus continues to work on improving the efficiency of nutrition to reduce GHG emissions. Based on the evidence presented above, we hypothesize that the use of FRC as a soybean meal replacement in broiler chicken diets may modulate cecal microbiota and consequently mitigate methane production, without affecting growth performance parameters, nitrogen retention, or ether extract digestibility. The present study assumes that FRC can improve the fatty acid (FA) profile of chicken breast. Hence, the aim of this study was to evaluate the effect of FRC on growth performance, nutrient utilization, methane emission, and breast meat FA composition in broiler chickens.

## 2. Materials and Methods

In line with Polish law and EU directive 2010/63/EU, the experiments conducted as part of this study did not require approval of the Local Ethical Committee for Experiments on Animals in Poznań.

### 2.1. Preparation of Fermented Rapeseed Cake

Rapeseed cake was fermented using a patent-pending (no. 422849) procedure described in details by Drażbo et al. [12]. Briefly, commercial rapeseed cake was ground and completely mixed with water at a ratio of 1:2 in plastic containers. A commercial 6-phytase enzyme preparation expressed by *Pichia pastoris* was used to ferment the rapeseed cake by adding it to the raw cake at a ratio of 1:1000 by weight basis and thoroughly mixing it. Solid-state fermentation was conducted for 24 h at 30 °C under anaerobic conditions. The enzyme was then deactivated at 70 °C for 15 min. The fermented biomass was dried at 55 °C.

### 2.2. Birds and Housing

Three independent experiments were carried out on the same batch of the fermented rapeseed cake and diet (Table 1). A total of 420 one-day-old female Ross 308 chicks (300 in experiment 1, 60 in experiment 2, and 60 in experiment 3) obtained from a local commercial hatchery (Ostrów Wielkopolski, Poland) were raised in a chicken house with a controlled environment. The first trial was conducted to explore the effects of raw rapeseed cake (RRC) or fermented rapeseed cake (FRC) added to the broiler chicken diets on their growth performance parameters, and the apparent total tract digestibility of ether extract and nitrogen retention. In total, 300 one-day-old female Ross 308 chicks were used and allotted to three groups, ten replicates each (ten birds in each replicate), according to a completely randomized design. The first experiment lasted from day 7 to day 35. To obtain the high uniformity of the birds, the start of the experimentation was set up on day 7. Due to the above, the birds were fed a basal diet (without the experimental factor) until d 7, and next the birds were randomly distributed to the homogenous groups with the average initial weight 164 ± 1 g. The birds were weighed on days 7, 14, 28, and 35. The body weight gain (BWG), feed intake (FI), and feed conversion ratio (FCR) were calculated for the 7–14 day, 15–28 day, 29–35 day, and 7–35 day periods. No mortality was observed in any of the treatment groups. Nitrogen retention and the apparent total tract digestibility of ether extract were analyzed using an internal marker (TiO_2_) from the bird’s excreta, which were collected on days 14 and 28, respectively. The fresh excreta from each pen (*n* = 10; ten birds’ excreta samples pooled within a pen represented one replication) were immediately frozen, lyophilized, ground, and stored at −20 °C for analysis. The birds were reared in floor pens (1.00 m × 1.00 m; straw litter) over 35 days on a density of 10 birds/m^2^. An artificial light program (fluorescent lights), forced ventilation, and automatic heaters were provided for the closed chicken barn. The lighting program was set up according to the recommendations of the Aviagen Management Handbook [13], and Council Directive (2007/43/EC). The birds were given 23 h of light and 1 h of dark during the 1st week of age, after that, from 7 to 32 days of age, they were provided 18 h of light and 6 h of dark. At last 3 days before the trial end there was 23 h light and 1 h of dark. In line with EU directive 2007/43/EC, the light intensity was set to 20 lx. The temperature was maintained at 32 °C on day 1 and was gradually reduced to 21 °C by day 21, at which it was maintained.

The second trial was conducted to explore the effects of RRC or FRC added to the diets on fourteen-day-old birds’ cecal anaerobic fermentation parameters (in vitro), and on the FA profiles of the breast tissue. A total of 60 one-day-old Ross 308 female chicks were randomly divided into three groups of 20 chicks, and there were ten replications per treatment and two birds per replication. All diets were applied as in the first trial and given ad libitum diet for two weeks (feed duration of 14 d after hatching). The regulated environmental conditions under which the chicks were raised were similar to those in the first trial. At the end of the experimental period (14 day), all animals were slaughtered and eviscerated. Their ceca were collected immediately and their contents were weighed for use in the in vitro fermentation experiment. The digesta from the cecum of one bird were too small to culture individually. Thus, following Tsukahara et al. [14], cecum digesta were pooled for two birds at a time. Thus, 5 g of cecal contents were weighed immediately into a 100-mL glass syringe (Häberle Labortechnik, Lonsee-Ettlenschieß, Germany) with 20 mL of anaerobic sodium phosphate buffer (50 mM, pH 6.5). The syringes were then filled with CO_2_, closed with rubber stoppers, and incubated in a rotating incubator (Przybylski, Poznań, Poland) at 39 °C for 7 h. The syringes were rotated automatically to allow the cecal contents to mix well with the buffer.

After chicken slaughter, breast muscle tissue was promptly packaged using flex-grip bags and stored at −80 °C for FA analysis.

The third trial aimed to explore the in vitro effects of RRC or FRC added to broiler chicken diets on anaerobic fermentation metrics of 28-day-old birds’ cecum contents, and on the FA profiles of their breast tissue. The experimental procedure was conducted similarly to that of the second trial, except that all diets were given ad libitum for four weeks (feed duration of 28 d after hatching).

### 2.3. Diets and Feeding Program

Table 1 presents the composition of the control and experimental diets. In all trials, the birds were fed ad libitum and had free access to drinking water. Reasonable nutrient requirements for broiler chickens were calculated to meet or exceed the NRC recommendations [15]. The use of viscous cereals (wheat) and animal dietary fat (pig lard) as ingredients was designed to provoke gastrointestinal tract colonization by *Clostridium perfringens* [16]. The mash-form diets were produced at Piast Pasze feed mill (Ostrów Wielkopolski, Poland), in accordance with ISO 9001:2008 procedures. A disc mill (Skiold, Sæby, Denmark) set with a 2.5-mm disc distance was used to grind all the raw materials. A laboratory-scale line equipped with a horizontal double band mixer (Zuptor, Gostyń, Poland) with roller mills (Skiold, Sæby, Denmark) was used to prepare the feed without the use of exogenous enzymes. The same basal diet (control group diet) was offered to all birds from day 1 to day 6 of age; the experimental diets were offered from day 7 to day 35 (experiment 1), day 7 to day 14 (experiment 2), or day 7 to day 28 (experiment 3). In all trials, the following treatments were used: CON: the basal diet without RRC or FRC; RRC: the basal diet with the inclusion of 150 g/kg RRC; and FRC: the basal diet with the inclusion of 150 g/kg FRC.

Table 2 shows the results of the feed samples analyzed for nonstarch polysaccharides (NSP) and glucosinolates, as partially reported by Drażbo et al. [8]. Gas−liquid chromatography (constituent neutral sugars) equipped with an SP-2340 column and a Varian CP3380 gas chromatograph (Varian, Palo Alto, CA, USA) were used to determine NSP. Uronic acids were determined, as described by Scott [17]. The sugars glucose, fructose, sucrose, raffinose, and stachyose were determined using the described by Slominski et al. [18]. Glucosinolates were analyzed by gas-liquid chromatography, as described by Slominski and Campbell [19].

### 2.4. Determination of the Anaerobic Fermentation Metrics of Cecal Contents

After 7 h anaerobic incubation, samples were taken immediately, filtrated, and analyzed. The pH values of the cecal content liquid samples were determined using a pH meter (CP-104, Elmetron, Zabrze, Poland). Ammonia concentration was analyzed using the protocol previously described by Szumacher-Strabel et al. [20]. Total gas production was measured directly using calibrated syringes. Methane concentration was measured following the procedure used by Szumacher-Strabel et al. [20]. Briefly, 1000 μL of gas sample was collected using GASTIGHT Syringes (Hamilton Bonaduz AG, Bonaduz, Switzerland) from the headspace of each syringe. The gas sample was then injected to the gas chromatograph (GC; SRI Peak Simple model 310, Alltech, Lexington, KY, USA). Ultra-high purity nitrogen gas (Air Products, Poznań, Poland) was used as the carrier gas at a constant flow of 30.0 mL/min. The oven temperature was programmed as follows: initially 180 °C for 1.5 min, then increasing at 20 °C/min to 220 °C. The GC was equipped with a thermal conductivity detector and a Carboxen 1000 column (mesh side 60/80, 15 FT × 1.8 INS.S, Supelco, Bellefonte, PA, USA). Methane concentration was quantified with an appropriate gas standard (mix gases of 5.63% CO_2_, 5.56% CH_4_, 5.0/d10% H_2_, and the rest N_2_ (Multax, Zielonki-Parcela, Warsaw, Poland)) using PeakSimple v. 3.29. The total bacteria and methanogens were determined by the fluorescent in situ hybridization (FISH) technique. The total bacteria and methanogens were analyzed following the procedure of Szczechowiak et al. [21] with modification; briefly, 100 μL of the buffered cecal content was diluted in PBS under ultrasonic oscillation (UP50H Compact Lab Homogenizer, Berlin, Germany) for ten seconds, and subsequently pipetted onto 0.22 μm polycarbonate filters (Frisenette, Knebel, Denmark) using a vacuum pump (KNF Neuberger, Freiburg, Germany). Next, cellulose discs were placed on the filters and immersed in increasing dilutions of ethanol for dehydration (50%, 80%, and 90%, 3 min each). Oligonucleotide probes (Archaea, S-D-Arch-0915-a-A-20; Sequence 5′ to 3′, GTGCTCCCCCGCCAATTCCT) were used to identify the methanogens with optimal hybridization used for this study [22]. Twenty-five microliters of hybridization buffer (5 M NaCl; 1 M Tris, 9.5 mL formamide; and 10% SDS) with 2.5 μL of the oligonucleotide probe was used for the hybridization; a washing buffer (1 M Tris, 5 M NaCl, and 80 mL H_2_O) was then used to wash the filters at 50 °C for 10 min. After washing, filters were cleaned gently in deionized water, air-dried, and inlaid on slides with VectaShield (Vector Laboratories, Burlingame, CA, USA), with one drop of antifading agent containing DAPI (4′,6-diamidino-2-phenylindole). So, as to distinguish bacteria from other particles in the cecal content, the filters were kept at 4 °C for 1 h in the dark prior to visualization with an Axio Imager M2 microscope (Carl Zeiss, Jena, Germany). For each polycarbonate filter, ten areas were measured (twenty fluorescent microscopy snapshots, two snapshots per area, including one snapshot of total bacteria and then a snapshot for the methanogens). The total bacterium and methanogen counts were made using ZEN 2.5 Pro software (Carl Zeiss Microscopy, Jena, Germany), using a mean of every ten measurements for each sample.

### 2.5. Chemical Analysis

The chemical compositions of the diets and the excreta were determined following AOAC methods [23] for determination of dry matter (DM; method no. 934.01), crude protein (CP; using a Kjel-Foss Automatic 16,210 analyzer (A/S N. Foss Electric, Hillerød, Dania); method no. 976.05), and ether extract (EE; using a Soxhlet System HT analyzer, Tecator, Honganas, Sweden; method no. 973.18). The gross energy was determined using an adiabatic bomb calorimeter (KL 12Mn, Precyzja-Bit, Poland) standardized with benzoic acid, following method PN-EN ISO 9831. Samples were prepared for titanium dioxide (TiO_2_) analysis, as previously reported by Myers et al. [24], and concentration was estimated by adapting the procedure used by Short et al. [25].

The FA concentration in breast tissue was determined using a gas chromatograph following Cieślak at al. [26]. Briefly, 700 mg of well-milled breast tissue was transferred into a screw-cap Teflon-stoppered tube (Pyrex, 15 mL). The hydrolyzation process began by adding 2 M NaOH (3 mL) to each tube at 90 °C for 40 min. Tubes were shaken vigorously every 10 min. Samples were then extracted using diethyl ether and were esterified to FA methyl esters (FAME) using boron trifluoride (Fluka-Sigma Aldrich, St. Louis, MO, USA). The 1 μL of sample was injected into a gas chromatograph (GC Bruker 456-GC, Billerica, MA, USA) equipped with 100-m fused-silica capillary column (0.25 mm ID) coated with 0.25 m Agilent HP (Chrompack CP7420) and a flame ionization detector. Temperatures in the injector and detector were maintained at 200 °C and 250 °C, respectively. The oven temperature was initially set at 120 °C for 7 min, then increased at a rate of 7 °C/min to 140 °C, held for 10 min, and then increased at a rate of 4 °C/min to 240 °C. Hydrogen gas was used as a carrier gas at a flow rate of 1.3 mL/min. The FAs were expressed as a proportion of the sum of identified FAs (g/100 g^−1^) and quantified with an appropriate FAME standard (37 FAME Mix, Sigma-Aldrich, Darmstadt, Germany) using Galaxie Work Station 10.1 software (Varian, Walnut Creek, CA, USA).

### 2.6. Calculations

(1) The *atherogenic* and *thrombogenic indexes* were calculated according to Ulbricht and Southgate [27] as follows:
  (1) $$Atherogenic\;index=\left(\frac{C12:0+4\;x\;C14:0+C16:0}{\sum MUFA+\sum \left(n–6\right)+\sum \left(n–3\right)}\right)$$
  (2) $$Thrombogenic\;index=\left(\frac{C14:0+C16:0+C18:0}{0.5\;x\;\sum MUFA+0.5\;x\;\sum \left(n–6\right)+3\;x\;\sum \left(n–3\right)+\frac{\sum \left(n–3\right)}{\sum \left(n–6\right)}}\right)$$
(2) The equations used to calculate the apparent total tract digestibility (*ATTD*) of ether extract (*EE*) and nitrogen retention relative to the ratio of titanium dioxide, according to Kierończyk et al. [28], are exemplified here by the equation for *EE* digestibility.
  (3) $$ATTD_{EE}=1-\left(\left(\frac{TiO_{2\;\frac{g}{kg}diet}}{TiO_{2\frac{g}{kg}excreta}}\right)\times \;\left(\frac{EE_{\frac{g}{kg}excreta}}{EE_{\frac{g}{kg}diet}}\right)\right)$$

### 2.7. Statistical Analysis

All the experiments had a completely randomized design. All data were tested for normal distributions using the Kolmogorov–Smirnov or Shapiro–Wilk test, depending on the data size. The animal pen was treated as the experimental unit for parameters, with respect to the effects of dietary treatment on growth performance parameters and ATTD, and nitrogen retention. In experiment 1, these were averaged for the pen. In experiments 2 and 3, for the in vitro anaerobic fermentation parameters (and total bacteria and methanogens), cecal digesta was pooled for two birds at a time into one sample; for the breast tissue FA parameters, ten birds from each group were randomly selected (experiments 2 and 3). Fatty acid analysis of breast tissue, anaerobic fermentation parameters (including total bacteria and methanogens), ATTD, nitrogen retention, BWG, FI, and FCR were determined by one-way analysis of variance (ANOVA) or the Kruskal–Wallis test, depending on whether normality of the data was confirmed or not. Data management and analysis were performed using SPSS 24.0 for Windows (IBM, Armonk, NY, USA). A pooled standard error of the mean (SEM) was used for the expressed variability of the data. The post-hoc Tukey test was used to determine differences among treatment groups at the significance level of *p* ≤ 0.05.

## 3. Results

### 3.1. Chemical Composition

The results presented in Table 2 have been partially published in Drażbo et al. [8] as a part of the same project, where the same batch of RRC and FRC was used. The FRC contained fewer glucosinolates than the RRC. The concentration of antinutritional compounds in rapeseed cake reduced with fermentation. The level of glucosinolates was 90% lower, and of carbohydrates 40.6% lower, in FRC than in RRC. The NSP concentration showed no difference between the RRC and FRC.

### 3.2. Fatty Acid Profile in the Diet

Experiments 1, 2, and 3: Table 3 provides the results of the analysis of FA profiles from the diets. With the inclusion of RRC and particularly of FRC, the concentrations of C18:1 *c*9 increased. The FRC group contained a higher level of total monounsaturated fatty acids (MUFAs) and unsaturated fatty acids (UFAs) than the RRC and CON diets, and a more desirable ratio of omega-6 to omega-3. There are very few differences between the groups in terms of particular polyunsaturated fatty acids (PUFAs). Additionally, the lowest value of total saturated fatty acids (SFAs) was found in the FRC group.

### 3.3. Growth Performance and Digestibility

Experiment 1: Table 4 shows the growth performance results. In the first (days 7–14), second (days 15–28), and third period (days 29–35), and during the entire experimental period (days 7–35), none of the between-group differences in BWG, FI, or FCR were statistically significant (*p* > 0.05).

The ATTD of the ether extract and nitrogen retention are shown in Table 5. No significant (*p* > 0.05) differences were observed among the treatments.

### 3.4. Cecal Content Fermentation Characteristics (In Vitro)

Experiment 2: The effect of RRC or FRC on the anaerobic fermentation parameters of cecal contents of 14-day-old birds is presented in Table 6. The total methanogens were significantly decreased (*p* < 0.001) in the FRC group, and methane production was consequently reduced (*p* = 0.027) in comparison with the CON group. The FRC group contained 65.8% fewer total methanogens and produced 20.83% less methane than the CON group. However, methanogens and methane production did not differ significantly between the RRC and CON groups. A positive relation was found between pH and methane concentration, with the lowest pH being observed in the FRC group; these differences were statistically significant (*p* = 0.001). Significantly less total gas production (TGP) was detected in the FRC group than in the other two groups (*p* = 0.003). RRC and FRC significantly decreased (*p* < 0.001) ammonia concentration and FRC significantly increased (*p* = 0.044) total bacteria counts.

Experiment 3: The results of the analysis of the fermentation parameters of cecal contents of 28-day-old birds are summarized in Table 7. There is a clear trend towards a significant reduction (*p* < 0.001) in methane production in the RRC and FRC groups compared with the CON group. A significant (*p* < 0.001) limitation in total methanogens was highlighted in the RRC and FRC groups. The pH value was significantly lower (*p* = 0.007) in the FRC group than in the CON group. Ammonia concentration decreased significantly (*p* < 0.001) in the RRC group and total bacteria was significantly higher in both RRC and FRC groups (*p* < 0.001), compared with the CON group.

### 3.5. Fatty Acid Profiles in the Breast Muscle

Experiment 2 (14-day-old birds): The FA composition of breast muscle is shown in Table 8. Broilers fed the RRC and FRC diets showed higher (*p* < 0.001) concentrations of myristic acid than the CON group. A reduction in stearic acid (*p* = 0.002) and behenic acid (*p* < 0.001) content in the breast muscle was achieved with the RRC and FRC diets. Consequently, total SFA decreased (*p* < 0.001) in the breast meat of the birds administered the RRC and FRC diets. Furthermore, broilers fed the RRC diet showed greater oleic acid contents (*p* = 0.05) than broilers offered the CON diet. Total MUFA increased in the breast meat of the birds administered with RRC diet (*p* = 0.025). A significantly lower level of eicosadienoic acid (*p* < 0.001) and EPA (*p* < 0.001) was seen in the birds on the RRC and FRC diets, while DHgL (*p* < 0.001) and DPA (*p* < 0.001) were lowered in the birds fed FRC, but not RRC, compared to broilers offered the CON diet. In contrast, γ-linolenic acid (*p* = 0.012) increased in the birds maintained on the FRC diet, but not on CON, compared with broilers offered the RRC diet. Moreover, a higher level of arachidonic acid (*p* < 0.001) was achieved by the FRC diet than in the other groups, and broilers offered the RRC diet had lower concentrations of DHA (*p* = 0.019) than the CON group. Linoleic acid (*p* < 0.001) and α-linolenic acid (*p* < 0.001) concentrations in breast muscle were significantly higher in FRC and RRC groups than in broilers offered the CON diet. Taken together, interestingly, the n-6/n-3 ratio (*p* = 0.018) increased in the birds that received RRC diet, but not in those receiving FRC, compared with CON. The ratio of PUFA:SFA (*p* = 0.027) increased in the birds that received the FRC diet but not the RRC diet, compared with CON. The use of RRC or FRC diet resulted in significantly lower (*p* < 0.001) thrombogenic indexes in broiler chickens than in the CON group.

Experiment 3 (28-day-old birds): The FA profile of 28-d broiler chickens breast muscle is presented in Table 9. A reduction in C14:0 (*p* < 0.001), C16:0 (*p* < 0.001), and C22:0 (*p* < 0.001) contents of the breast muscle was achieved when RRC and FRC were incorporated to the diets; consequently, total SFA (*p* < 0.001) decreased in the breast muscle of the birds administered the RRC and FRC diets. The level of C18:1 *c*11 (*p* < 0.001) in the breast muscle of the RRC and FRC groups was significantly higher than in the CON group; on the other hand, the level of C18:1 *c*9 (*p* = 0.002) was significantly greater in the FRC group, but not the RRC group, than in the CON. Hence, total MUFA increased in the breast muscle of the birds administered the RRC and FRC diets (*p* < 0.001). Moreover, breast muscle levels of DHgL (*p* < 0.001) and EPA (*p* < 0.001) were significantly reduced with the RRC and FRC diets. In contrast, C18:3 *c*9*c*12*c*15 (*p* < 0.001) and C20:2 (*p* < 0.001) levels significantly increased with the RRC and FRC diets, as compared to CON. The level of C22:6 n-3 (*p* < 0.001) was higher in the birds maintained on the RRC diet, but not on FRC, than in broilers offered the CON diet; however, the level of C18:3 n-6 (*p* = 0.024) was higher in the birds maintained on the FRC diet (though not on the CON diet) than in broilers offered the RRC diet. Broilers fed the RRC diet showed increased levels of C20:4 n-6 (*p* = 0.025) than those on FRC, though not CON. Our results show that the total LCFA content was significantly higher (*p* < 0.001) in both the RRC and FRC groups than in the CON group. All the tested diets significantly decreased omega-6 fatty acids (*p* < 0.001), the n-6/n-3 ratio (*p* < 0.001), whereas the ratio of PUFA:SFA (*p* = 0.024) increased in the birds that received the FRC diet, but not the RRC diet, compared with CON. The use of the FRC diet significantly reduced the atherogenic index (*p* = 0.004) and thrombogenic index (*p* = 0.008) in broiler chickens, compared with the CON group.

## 4. Discussion

The use of crude rapeseed cake in broiler chicken diets has been frequently studied. The effect of this raw material on performance depends on the amount supplied. Thacker et al. [29] confirmed that green canola cakes fed making up 15% of the broiler chicken diet did not significantly affect BWG or FI during days 0–21 of the feeding period. Moreover, Smulikowska et al. [30] have shown that the use of RRC in broiler chicken diets resulted in slightly reduced FI and BWG over the 42 days of feeding. The high level of crude fiber and antinutritional factors (such as glucosinolates) are potential explanations of this. Isothiocyanates are produced through the hydrolyzation of glucosinolates, with the participation of myrosinase, leading to a reduction in the FI, and thus an adverse impact on the BWG [31].

Fermentation has thus been proposed to improve the nutritional characteristics of fermented rapeseed products [32], although no information regarding changes in fatty acid profiles was found. According to Goodarzi Boroojeni et al. [33], fermentation can improve the nutritional value of plant protein feeds and reduce the concentration of glucosinolates, increasing the total protein content of rapeseed feedstuffs [34,35]. This is confirmed by the present study, where glucosinolates, gluconapin, glucobrassicinapin, progoitrin, hydroxyglucobrassicin, and sucrose were reduced by the fermentation process. However, there was no effect of FRC on the growth performance or on the nutrient utilization of broiler chickens, which is consistent with results of Chiang et al. [34] and Xu et al. [36]. Nevertheless, Ashayerizadeh et al. [37] showed the greatest beneficial effect when 50% of soybean meal was replaced by FRSM (153.6 g/kg). Further research is thus recommended to confirm the beneficial effects of the fermentation process. However it should be stressed that increasing rapeseed cake in broiler chicken diets by replacement of soybean meal should be considered as an important factor in terms of locally produced proteins usage. Particularly in countries that are dependent on soybean import.

The fatty acid composition of meat products plays a pivotal role in human health [38]. Aziza et al. [39] reported that diet can modify chicken meat quality, and Parveen et al. [40] confirmed that the level of benefits to human health of PUFAs in tissues of birds is associated with diet. A strong relationship has been reported between decreased dietary intake of n-3 PUFAs, high n-6/n-3 ratio, and the development of cardiovascular and heart diseases, and various malignant diseases [7]. Dietary SFAs (like myristic, palmitic, and stearic acid), due to their hypercholesterolemic properties, play an important role in the development of coronary heart disease [41]. On the other hand, the long-chain fatty acids (LCFAs) are advantageous in preventing metabolic disorders [42]. The present study has shown that the SFAs in the birds’ breast muscle tissue were affected by the dietary treatments, however, no significant changes were observed between the experimental groups, which indicates that it was not the fermentation process that had an effect on the results, but the diet (rapeseed cake) used. LCFA concentrations were higher when RRC and FRC diets were offered. In connection with fermentation, comparison of the findings with those of the other study confirms that FRC used in broiler chicken diets to replace up to 50% of soybean meal may elevate breast muscle concentrations of C18:2, C18:3, and C20:4, and total MUFA, while decreasing total SFA [37]. Atherogenic and the thrombogenic indices should be as low as possible in health-promoting diets [27]. In the present study, the FRC diet reduced both indices in comparison with the CON diet (experiment 3), although in most of the analyzed FA (except C18:1 *c*9) there were no changes compared to RRC. The literature has only limited data on the effect of FRC diets on the meat FA profile in poultry. In the experiment of Bedeković et al. [7], the inclusion of 10% rapeseed cake to broiler chicken diets increased the levels of omega-3, including DHA, and decreased the omega-6:omega-3 ratio in older birds’ breast muscle. This finding is consistent with our results for the profile of 28-day broiler breast meat when RRC and FRC were used. The results of this study confirm the association between raw or fermented rapeseed byproducts fed to broilers and the FA profile, including the SFA and UFA concentrations in broiler breast muscle, supporting the findings of previous observations [37] and further demonstrating that FRC and RRC can reduce myristic acid, palmitic acid, and stearic acid in the breast muscle in a way dependent on the age of the birds. As mentioned above, high UFA and low SFA levels contribute to the attractiveness of poultry breast meat to consumers, as they provide protection against cardiovascular diseases. Independently of the type of animal used in the experiment, higher concentrations of n-3 and lower n-6/n-3 ratios were observed in the experimental groups, which were fed diet supplemented with 15% raw rapeseed and fermented rapeseed meal [8]. Previous research on using rapeseed products in poultry showed increased amounts of n-3 in the meat [7,43,44]. These results corroborate the findings that rapeseed oil or rapeseed byproducts, such as meal or cake, could enhance the n-3 concentration of the poultry product, as we observed in the present study with broiler chickens fed both RRC and FRC. The RRC and FRC diets improved the n-6/n-3 ratio, which is advantageous for obtaining healthy chicken products. This effect was found in 28-d birds. These differences may be explained by age [45]. A positive effect on the fatty acid profile was obtained using both RRC and FRC. The advantage of using FRC is due to the reduced content of antinutritional factors, which would allow for wide application in poultry nutrition in the future.

More than 65% of global atmospheric methane emissions originate from anthropogenic sources. Overall, animal production emits 90 million tonnes of methane per year. In the case of ruminants, most of the methane and other gases produced and emitted comes from intestinal fermentation (80%), while in the case of poultry it comes either from intestinal fermentation or from fermentation of stored manure [46]. In many countries and regions, data are collected on the size of the emission of gases from the animal production, which consequently leads to the development of rules on balanced ecodevelopment [47]. According to data from the National Inventory Report [48] on the size of production of GHGs in Poland over the years 1988–2017, and according to our own unpublished data, the index of methane emission is 97 kg/dairy cow/year and 1.5 kg/pig/year, while for poultry it is on average 36 g/bird/year; these data concern only enteric fermentation. Taking into account the almost one billion chickens and counting methane emission only from intestinal fermentation (discounting emissions from manure), we conclude that the total annual production of methane in Poland amounts to approximately 36 thousand tonnes per year. For comparison, pigs in Poland produce 16 thousand tonnes annually. The problem gains weight in the face of continually rising environmental requirements from the European Union. Indications are that the majority of GHG emissions generated from the poultry industry occur during the production stage. The highest proportion (48.3%) of emissions came from broiler farms rather than breeder farms. It is therefore important that the poultry industry continues to work on improving the efficiency of nutrition so as to reduce GHG emissions. The global poultry sector is vast in size, hence there is a necessity to be vigilant and to begin working to reduce emissions and make the industry more sustainable. It is worth highlighting that the IPCC guide to national GHG inventories [49] still does not take domestic poultry enteric methane emission into account. There have been some studies on the methane generated from broiler chickens’ cecal content and from other poultry species [50,51], which should be included in the report. The present research is one of the first to examine the effects of dietary composition on the level of methane emissions from broiler chickens, and our results can enrich the worldwide database.

As our literature review mentioned, unlike in ruminants, the main fermentation site in poultry is the ceca, and this is where fermentation byproducts, such as H_2_ and CO_2_, are used by methanogens to produce methane [51,52,53]. We observed a reduction in methane emission when FRC was fed to the birds, regardless of age, and this has the direct effect of limiting the number of methanogens. Little information is available on the production of methane from poultry ceca; however, existing data indicate the effect of the age of birds on the amount of methane produced. Young mule ducks, Muscovy goslings, and White Roman goslings generated less methane from cecal content than older birds, which has been explained by changes in nutrient metabolism in the intestinal tract of birds [54]. The results of the present study corroborate the ideas of Chen et al. [54] and Xia et al. [55], who suggested changes in methanogen population with age. Moreover it is well documented that it takes up to 65 days to establish relatively stable microbiome in ceca, and from 14 to 28 d many changes particularly in *Clostridiacae* are observed. Additionally, one review noted the importance of fermented feeds in poultry gastrointestinal ecosystems, giving major benefits such as lowered pH and high numbers of lactobacilli [34,50,53,56,57]. In the present study, mitigation of methane production was observed when FRC was fed either to 14-d or 28-d old birds, but the concentration of cecal methanogens was higher (23% when the control group was compared) in older chickens. The amount of methane emission reduction depends on the age of the birds. For the FRC diet, within the first 14 d, there was a 21% reduction in methane output, whereas within 28 days a 52% reduction in methane output was observed.

Cecal fermentation and the associated CH_4_ production is related to the microbial digestion of uric acid, which may partly explain our finding of methane emission from CON and RRC or FRC groups, regardless of their age [51,58]. Furthermore, the results for ammonia are in agreement with Chen et al. [59] and with Abdl-Rahman et al. [60], who stated that higher cecal ammonia levels resulted in an increased pH. Rea et al. [61] showed that the optimal growth conditions for methanogens are in the range of 6.0–7.5 pH, and that decreases in pH adversely affect methanogens. These relationships might partly explain why there were fewer methanogens in the FRC group. As summarized in Misiukiewicz et al. [62], literature data suggest that there are about 10^5^–10^7^ cells of Archaea of all types per gram of cecal material on a wet weight basis. Moreover, Archaea of all types constitute approximately 1% of the total microbial count in the ceca, which is in agreement with the findings of the present study in the CON groups from both experiments (0.72 and 0.83, respectively in 14-d and 28-d old birds cecal material). Feed form and quality determine the processes that occur in the ceca. Fermentation processes improve the efficiency of feed utilization by animals in the gastrointestinal tract [53,63], and less methane may consequently be generated than with an unfermented diet, as has been confirmed in the current study. To the best of our knowledge, the effect of FRC on the in vitro methane production of broiler chickens has not yet been investigated, and our observations may support the above hypothesis. We are aware of the fact that our research has some limitations, e.g., the experiment was conducted in vitro and the incubated material was cecum, which, due to changes in the number of microorganisms, is a very dynamically changing environment. Thus, the authors realize that the interpretation of the obtained results should be carried out with caution. However, the small amount of data in the literature on methane production from chickens prompts us to present the results as important in terms of understanding this process in poultry. The planned further research will include an aspect of methane emission analysis using respiration chambers. Moreover, the population of methanogens might also decrease due to the presence of NSP in the diet [64]. However, according to Marounek et al. [65], different in-feed substrates (lactose, raffinose, starch, inulin, pectin, xylan, and cellulose) did not affect CH_4_ production from cecal contents of chickens, and altered only minor fermentation patterns. Our findings broadly agree with the results of other studies in this area presenting an increased population of microorganisms [66,67]. The bacterial population increased in the RRC and FRC groups in both experiments, but the highest increase was seen for the FRC groups in experiments 2 and 3. The increased cecal population of Lachnospiraceae*, Lactobacillus* spp.*, Streptococcus* spp.*, Bacteroides* spp.*,* and Alphaproteobacteria as birds age may contribute to the increased final total bacteria population [55,68].

## 5. Conclusions

Our results show that soybean meal can be partially replaced by RRC and FRC without detrimental effects on the birds’ growth performance, ATTD of ether extract coefficient, or nitrogen retention. Including RRC and FRC in the diet decreased the n-6/n-3 ratio in chicken breast meat, resulting in a healthier meat product. This study has also identified the methane-mitigating effect of dietary FRC caused by the decreased cecal pH and consequently reduced methanogen population. Reduction of methane production by 21% and 51%, respectively in 14-d old and 28-d old broiler chickens, results in lower methane emission indices, at 7.56 g g/bird/year and 18.36 g/bird/year, depending on age. Therefore the addition of 15% FRC to the diet compared to inclusion RRC in diet is not only important in terms of locally grown sources of protein but also desirable from the environmental and human health point of view.

## Figures and Tables

**Table 1:** Composition and nutritive value of diets in experiments 1, 2, and 3.

Ingredient, g·kg^−1^	Days 7–35	
	CON ^a^	RRC/FRC ^b^
Wheat	580	499.4
Maize	100	100
Soybean meal 46.8%	228	157.2
Pig lard	44.6	52.7
Soybean oil	23	19.7
RRC or FRC ^b^		150
Mineral–vitamin premix ^c^	3	3
Monocalcium phosphate	1.7	0.5
Limestone	8.2	7.2
Salt (NaCl)	1.7	2
Sodium sulfate (Na_2_SO_4_)	1.9	1.6
L-Lysine	3.3	3.2
L-Methionine	2.2	1.8
L-Threonine	1.9	1.5
L-Valine	0.5	0.2
Calculated nutritive value, g·kg^−1^		
AME_N_ (MJ/kg) ^d^	13.52	13.52
Crude protein	190.0	195.0
Crude fat	82.8	96.8
Crude fiber	23.8	36.7
Calcium (Ca)	6.5	6.5
Lysine	11.2	11.5
Methionine + cysteine	8.6	8.8
Analyzed chemical composition, g·kg^−1^		
Gross energy (MJ·kg^−1^)	18.25	18.84/18.85
Crude protein	193.6	198.6/195.2
Ether extract	79.80	95.50/96.35

^a^ Control diet. ^b^ Raw rapeseed cake or fermented rapeseed cake. ^c^ Provided the following per kilogram of diet: vitamin A: 11,166 IU; cholecalciferol: 2500 IU; vitamin E: 80 mg; menadione: 2.50 mg; B_12_: 0.02 mg; folic acid: 1.17 mg; choline: 379 mg; d-pantothenic acid: 12.50 mg; riboflavin: 7.0 mg; niacin: 41.67 mg; thiamine: 2.17 mg; d-biotin: 0.18 mg; pyridoxine: 4.0 mg; ethoxyquin: 0.09 mg; Mn (MnO_2_): 73 mg; Zn (ZnO): 55 mg; Fe (FeSO_4_): 45 mg; Cu (CuSO_4_): 20 mg; I (CaI_2_O_6_): 0.62 mg; Se (Na_2_SeO_3_): 0.3 mg. ^d^ Apparent metabolizable energy corrected to zero nitrogen balance.

**Table 2:** Chemical composition of raw rapeseed cake (RRC) and fermented rapeseed cake (FRC) ^a^ in experiments 1, 2, and 3.

Component	RRC	FRC
Nonstarch polysaccharides (%, as-is basis)	22.25	22.57
NSP component sugars:		
Rhamnose	0.22	0.25
Arabinose	4.35	4.36
Xylose	1.76	1.83
Mannose	0.39	0.40
Galactose	1.49	1.53
Glucose	7.00	7.30
Uronic acids	7.03	6.90
Glucosinolates (µmol/g, as-is basis)	16.30	1.66
Gluconapin	3.09	0.29
Glucobrassicinapin	1.20	0.10
Progoitrin	8.89	0.54
Glucobrassicin	0.17	0.00
Hydroxyglucobrassicin	2.95	0.74
Sugars (%, as-is basis)	9.22	5.48
Simple sugars ^b^	0.51	0.41
Sucrose	5.85	2.85
Oligosaccharides ^c^	2.86	2.22

^a^ Results have been partially published in Drażbo et al. [8] as a part of the same project, where the same batch of RRC and FRC (Raw rapeseed cake or fermented rapeseed cake) was used; ^b^ includes glucose and fructose; ^c^ includes raffinose and stachyose.

**Table 3:** Fatty acid profiles of diets containing raw rapeseed cake (RRC) and fermented rapeseed cake (FRC) fed to the broiler chickens (g/100 g FA); experiments 1, 2, and 3.

Item	CON ^1^	RRC ^2^	FRC ^3^
Saturated			
C8:0 Caprylic	0.12	0.11	0.09
C10:0 Capric	0.08	0.14	0.13
C14:0 Myristic	0.45	0.43	0.42
C16:0 Palmitic	10.15	9.56	9.32
C18:0 Stearic	4.42	4.22	4.25
C20:0 Arachidonic	0.13	0.12	0.09
Monounsaturated			
C16:1 Palmitoleic	0.06	0.08	0.09
C18:1 *c*9 Oleic	24.04	24.79	26.00
C18:1 *c*11 Elaidic	1.97	2.60	2.19
C20:1 Eicosenoic *trans*	0.38	0.42	0.44
Polyunsaturated			
C18:2 *c*9*c*12 Linoleic, LA	47.57	47.28	46.37
C18:3 *c*9*c*12*c*15 α-Linolenic, LNA	7.36	7.69	7.47
SFA ^4^	16.40	15.40	15.33
UFA ^5^	83.60	84.60	84.67
MUFA ^6^	27.28	28.47	29.37
PUFA ^7^	56.32	56.14	55.30
Omega-6	48.79	48.33	47.58
Omega-3	7.53	7.81	7.73
Omega-6:Omega-3	6.48	6.19	6.16
PUFA:SFA	3.44	3.65	3.61
LNA:LA	0.15	0.16	0.16

^1^ control diet; ^2^ diet containing 15% raw rapeseed cake; ^3^ diet containing 15% fermented rapeseed cake; ^4^ saturated fatty acids; ^5^ unsaturated fatty acids; ^6^ monounsaturated fatty acids; ^7^ polyunsaturated fatty acids.

**Table 4:** Effects of raw or fermented rapeseed cake on the growth performance of broiler chickens; experiment 1.

Period	Treatment			SEM ^4^	*p*-Value
	CON ^1^	RRC ^2^	FRC ^3^		
BWG ^5^, g					
7–14 d	203	203	202	2.28	0.998
15–28 d	1105	1049	1066	15.32	0.328
29–35 d	628	641	690	16.23	0.268
7–35 d	1935	1893	1958	24.00	0.548
FI ^6^, g					
7–14 d	313	308	309	1.66	0.435
15–28 d	1490	1431	1431	15.78	0.214
29–35 d	985	969	1026	17.68	0.415
7–35 d	2788	2708	2766	26.57	0.462
FCR ^7^, g:g					
7–14 d	1.54	1.52	1.53	0.01	0.662
15–28 d	1.35	1.36	1.34	0.01	0.699
29–35 d	1.57	1.51	1.49	0.02	0.135
7–35 d	1.44	1.43	1.42	0.01	0.353

^1^ control diet; ^2^ diet containing 15% raw rapeseed cake; ^3^ diet containing 15% fermented rapeseed cake; ^4^ standard error of the mean; ^5^ body weight gain; ^6^ feed intake; ^7^ feed conversion ratio; means represent ten pens (*n* = 10, 10 birds per each pen).

**Table 5:** Effects of use of raw or fermented rapeseed cake on the coefficients of total tract apparent digestibility of ether extract and nitrogen retention in broiler chickens; experiment 1.

Item	Treatment			SEM ^4^	*p*-Value
	CON ^1^	RRC ^2^	FRC ^3^		
N ^5^, %					
day 14	63.22	63.12	63.93	0.59	0.846
day 28	59.17	60.55	59.96	0.79	0.783
EE ^6^, %					
day 14	91.07	91.70	92.47	0.34	0.265
day 28	92.68	91.30	92.63	0.49	0.431

^1^ control diet; ^2^ diet containing 15% raw rapeseed cake; ^3^ diet containing 15% fermented rapeseed cake; ^4^ standard error of the mean; ^5^ nitrogen retention; ^6^ coefficients of total tract apparent digestibility of ether extract; means represent ten pens.

**Table 6:** Effect of diets containing raw rapeseed cake (RRC) and fermented rapeseed cake (FRC) on fermentation parameters (mean ± SD) in the ceca of broiler chickens (14-day-old birds); experiment 2.

Parameters	Treatment									SEM ^4^	*p*-Value
	CON ^1^			RRC ^2^			FRC ^3^				
pH	5.79 ^a^	±	0.25	5.64 ^ab^	±	0.14	5.45 ^b^	±	0.03	0.041	0.001
NH_3_ (mM)	12.31 ^a^	±	0.64	7.79 ^c^	±	1.00	9.91 ^b^	±	0.96	0.397	<0.001
TGP (mL)	5.33 ^a^	±	0.71	5.00 ^a^	±	0.87	3.67 ^b^	±	0.87	0.207	0.003
CH_4_ (μM)	2.40 ^a^	±	0.16	2.00 ^ab^	±	0.58	1.90 ^b^	±	0.30	0.083	0.027
Microbial populations											
Total methanogens (10^7^ mL^−1^)	3.77 ^a^	±	0.69	3.59 ^a^	±	0.29	1.25 ^b^	±	0.35	0.242	<0.001
Total bacteria (10^9^ mL^−1^)	5.27 ^b^	±	0.33	5.44 ^ab^	±	0.53	5.85 ^a^	±	0.52	0.099	0.044

^a–c^ Means not sharing a common superscript differ significantly (*p* < 0.05); ^1^ control diet; ^2^ diet containing 15% raw rapeseed cake; ^3^ diet containing 15% fermented rapeseed cake; ^4^ standard error of the mean; means represent ten pens.

**Table 7:** Effect of diets containing raw rapeseed cake (RRC) and fermented rapeseed cake (FRC) on fermentation parameters (mean ± SD) in the ceca of broiler chickens (28-day-old birds); experiment 3.

Parameters	Treatment									SEM ^4^	*p*-Value
	CON ^1^			RRC ^2^			FRC ^3^				
pH	5.81 ^a^	±	0.10	5.75 ^ab^	±	0.13	5.57 ^b^	±	0.14	0.031	0.007
NH_3_ (mM)	13.91 ^a^	±	0.49	8.68 ^b^	±	1.57	13.84 ^a^	±	2.79	0.638	<0.001
TGP (mL)	8.67	±	1.51	8.00	±	1.35	7.50	±	1.12	0.257	0.260
CH_4_ (μM)	14.4 ^a^	±	2.82	10.75 ^b^	±	2.63	6.95 ^c^	±	1.52	0.747	<0.001
Microbial populations											
Total methanogens (10^7^ mL^−1^)	4.68 ^a^	±	0.26	4.3 ^b^	±	0.36	2.5 ^c^	±	0.08	0.2	<0.001
Total bacteria (10^9^ mL^−1^)	5.63 ^b^	±	0.34	6.15 ^a^	±	0.26	6.43 ^a^	±	0.23	0.083	<0.001

^a–c^ Means not sharing a common superscript differ significantly (*p* < 0.05); ^1^ control diet; ^2^ diet containing 15% raw rapeseed cake; ^3^ diet containing 15% fermented rapeseed cake; ^4^ standard error of the mean; means represent ten pens.

**Table 8:** Effect of diets containing raw rapeseed cake (RRC) and fermented rapeseed cake (FRC) on fatty acid profiles of breast muscle (g/100 g FA; 14-day-old birds); experiment 2.

FA ^1^, (g/100 g)	Treatment			SEM ^5^	*p*-Value
	CON ^2^	RRC ^3^	FRC ^4^		
Saturated					
C14:0 Myristic	0.189 ^b^	0.325 ^a^	0.325 ^a^	0.013	<0.001
C16:0 Palmitic	17.78	17.25	17.44	0.113	0.178
C18:0 Stearic	14.39 ^a^	13.00 ^b^	13.26 ^b^	0.174	0.002
C20:0 Eicosanoic	0.094	0.093	0.108	0.004	0.197
C22:0 Behenic acid	1.731 ^a^	1.257 ^b^	1.368 ^b^	0.038	<0.001
Monounsaturated					
C16:1 Palmitoleic	0.319	0.320	0.314	0.006	0.922
C18:1 *c*9 Oleic	20.94 ^b^	23.08 ^a^	22.62 ^ab^	0.375	0.050
C18:1 *c*11 Elaidic	4.824	5.069	4.858	0.041	0.053
C20:1 Eicosenoic *trans*	0.077	0.071	0.078	0.003	0.670
C24:1 Nervonic acid	2.089	1.961	1.799	0.053	0.058
Polyunsaturated					
C18:2 *c*9*c*12 Linoleic	20.38 ^b^	22.44 ^a^	22.40 ^a^	0.183	<0.001
C18:3 *c*9*c*12 *c*15 α-linolenic	0.746 ^b^	1.080 ^a^	1.257 ^a^	0.049	<0.001
C18:3 n-6 γ-linolenic	0.487 ^ab^	0.446 ^b^	0.509 ^a^	0.008	0.012
C20:2 Eicosadienoic acid	0.337 ^a^	0.234 ^b^	0.233 ^b^	0.010	<0.001
C20:3 n-6 DHgL ^6^	9.045 ^a^	8.183 ^a^	7.083 ^b^	0.183	<0.001
C20:4 n-6 Arachidonic	0.126 ^b^	0.120 ^b^	0.168 ^a^	0.006	<0.001
C20:5 n-3 EPA ^7^	1.899 ^a^	1.662 ^b^	1.534 ^b^	0.035	<0.001
C22:5 n-3 DPA ^8^	1.667 ^a^	1.527 ^ab^	1.354 ^b^	0.038	<0.001
C22:6 n-3 DHA ^9^	0.019 ^a^	0.005 ^b^	0.016 ^ab^	0.002	0.019
Others ^10^	2.751 ^a^	2.415 ^b^	2.682 ^ab^	0.053	0.034
SFA ^11^	36.34 ^a^	34.33 ^b^	34.82 ^b^	0.189	<0.001
UFA ^12^	63.66 ^b^	65.67 ^a^	65.18 ^a^	0.189	<0.001
MUFA ^13^	28.74 ^b^	30.69 ^a^	28.90 ^ab^	0.309	0.025
PUFA ^14^	34.92	34.98	35.67	0.280	0.462
Omega-6	30.36	30.75	30.74	0.218	0.707
Omega-3	4.331	4.070	4.134	0.057	0.154
Omega-6:Omega-3	7.055 ^b^	7.610 ^a^	7.467 ^ab^	0.086	0.018
LCFAs ^14^	81.54	82.00	81.79	0.121	0.358
PUFA:SFA	0.970 ^b^	1.019 ^ab^	1.027 ^a^	0.01	0.027
Atherogenic index	0.295	0.289	0.288	0.002	0.351
Thrombogenic index	1.031^a^	0.957^b^	0.964^b^	0.007	<0.001

^a,b^ Means not sharing a common superscript differ significantly (*p* < 0.05); ^1^ fatty acid; ^2^ control diet; ^3^ diet containing 15% raw rapeseed cake; ^4^ diet containing 15% fermented rapeseed cake; ^5^ standard error of the mean; ^6^ dihomogammalinolenic acid; ^7^ eicosapentaenoic acid; ^8^ docosapentaenoic acid; ^9^ docosahexaenoic acid; ^10^ saturated (C8:0; C10:0; C12:0; C15:0; C17:0; C21:0; C23:0; C24:0), monounsaturated (C14:1; C18:1 *c*11; C18:1 *c*12; C22:1 n-9), and polyunsaturated (C22:2) fatty acids; ^11^ saturated fatty acids; ^12^ unsaturated fatty acids; ^13^ monounsaturated fatty acids; ^14^ polyunsaturated fatty acids; means represent ten pens.

**Table 9:** Effect of diets containing raw rapeseed cake (RRC) and fermented rapeseed cake (FRC) on fatty acid profiles of chicken breast muscle (g/100 g FA; 28-day-old birds); experiment 3.

FA ^1^, (g/100 g)	Treatment			SEM ^5^	*p*-Value
	CON ^2^	RRC ^3^	FRC ^4^		
Saturated					
C14:0 Myristic	0.847 ^a^	0.740 ^b^	0.688 ^b^	0.017	<0.001
C16:0 Palmitic	21.54 ^a^	20.36 ^b^	20.09 ^b^	0.102	<0.001
C18:0 Stearic	11.49	10.94	10.58	0.161	0.075
C20:0 Eicosanoic	0.102	0.091	0.107	0.003	0.088
C22:0 Behenic acid	0.825 ^a^	0.597 ^b^	0.540 ^b^	0.028	<0.001
Monounsaturated					
C16:1 Palmitoleic	0.502	0.536	0.525	0.010	0.410
C18:1 *c*9 Oleic	28.76 ^b^	30.85 ^ab^	32.29 ^a^	0.407	0.002
C18:1 *c*11 Elaidic	4.613 ^b^	5.243 ^a^	5.404 ^a^	0.055	<0.001
C20:1 Eicosenoic *trans*	0.087	0.080	0.082	0.002	0.501
C24:1 Nervonic acid	1.177	1.152	1.308	0.067	0.572
Polyunsaturated					
C18:2 *c*9*c*12 Linoleic	18.04	18.38	18.51	0.150	0.454
C18:3 *c*9*c*12 *c*15 α-linolenic	1.257 ^b^	1.693 ^a^	1.816 ^a^	0.056	<0.001
C18:3 n-6 γ-linolenic	0.410 ^ab^	0.409 ^b^	0.437 ^a^	0.005	0.024
C20:2 Eicosadienoic acid	0.234 ^b^	2.942 ^a^	2.414 ^a^	0.283	<0.001
C20:3 n-6 DHgL ^6^	4.765 ^a^	1.972 ^b^	1.073 ^b^	0.332	<0.001
C20:4 n-6 Arachidonic	0.081 ^ab^	0.108 ^a^	0.082 ^b^	0.005	0.025
C20:5 n-3 EPA ^7^	1.344 ^a^	0.869 ^b^	0.762 ^b^	0.046	<0.001
C22:5 n-3 DPA ^8^	0.724	0.811	0.769	0.035	0.634
C22:6 n-3 DHA ^9^	0.007 ^b^	0.022 ^a^	0.009 ^b^	0.002	<0.001
Others ^10^	2.274 ^a^	2.187 ^ab^	1.889 ^b^	0.062	0.025
SFA ^11^	36.21 ^a^	34.06 ^b^	33.27 ^b^	0.243	<0.001
UFA ^12^	63.79 ^b^	65.94 ^a^	66.73 ^a^	0.243	<0.001
MUFA ^13^	35.97 ^b^	38.56 ^a^	39.98 ^a^	0.359	<0.001
PUFA ^14^	27.37	27.38	26.98	0.185	0.603
Omega-6	24.31 ^a^	21.16 ^b^	21.01 ^b^	0.345	<0.001
Omega-3	3.384	3.433	3.443	0.032	0.762
Omega-6:Omega-3	6.969 ^a^	6.205 ^b^	6.183 ^b^	0.098	<0.001
LCFAs ^14^	76.83 ^b^	78.11 ^a^	78.44 ^a^	0.124	<0.001
PUFA:SFA	0.778 ^b^	0.805 ^ab^	0.818 ^a^	0.006	0.024
Atherogenic index	0.387 ^a^	0.372 ^ab^	0.361 ^b^	0.003	0.004
Thrombogenic index	1.057 ^a^	1.018 ^ab^	0.977 ^b^	0.010	0.008

^a,b^ Means not sharing a common superscript differ significantly (*p* < 0.05); ^1^ fatty acid; ^2^ control diet; ^3^ diet containing 15% raw rapeseed cake; ^4^ diet containing 15% fermented rapeseed cake; ^5^ standard error of the mean; ^6^ dihomogammalinolenic acid; ^7^ eicosapentaenoic acid; ^8^ docosapentaenoic acid; ^9^ docosahexaenoic acid; ^10^ saturated (C8:0; C10:0; C12:0; C15:0; C17:0; C21:0; C23:0; C24:0), monounsaturated (C14:1; C18:1 *c*11; C18:1 *c*12; C22:1 n-9), polyunsaturated (C22:2); ^11^ saturated fatty acids; ^12^ unsaturated fatty acids; ^13^ monounsaturated fatty acids; ^14^ polyunsaturated fatty acids; means represent ten pens.
